# An Atypical Presentation of Serotonin Syndrome

**DOI:** 10.7759/cureus.13377

**Published:** 2021-02-16

**Authors:** Eric Landa, Stephen Wagner, Abilash Makkar, Angdi Liu, Diana Jung

**Affiliations:** 1 Internal Medicine, Unity Health, Searcy, USA

**Keywords:** serotonin syndrome, lithium toxicity, serotonin toxicity

## Abstract

The purpose of this paper is to highlight an uncommon presentation of serotonin syndrome and discuss important points such as causes, the manifestation of symptoms, and available treatments. The report highlights the importance of recognizing typical signs and symptoms in order to uncover an atypical presentation of serotonin syndrome. Serotonin toxicity can become life-threatening if not identified early in its course and the offending agents discontinued. This can be achieved by educated physicians and careful prescribing of these agents.

## Introduction

Serotonin syndrome is caused by an excess of serotonergic agonism at receptors in both the central and peripheral nervous system, leading to the manifestation of a wide variety of symptoms from very mild to life-threatening. Identifying it early in its course can prove to be life-saving. But, what if a physician encounters a patient that does not quite fit the picture of serotonin toxicity? Herein, we present a case of a 67-year-old female with an atypical presentation of serotonin syndrome due to concurrent lithium toxicity. 

## Case presentation

A 67-year-old female with a significant medical history of depression and bipolar disorder (last manic episode in July) presented to the emergency department (ED) with her husband due to a fall. The history was obtained from the husband as the patient was lethargic and confused. Over the past month, she had been experiencing worsening depression, along with two episodes of falling due to weakness. During one of the episodes, she hit her head on the bathroom floor and began to bleed. Home medications included fluoxetine 20mg once a day, escitalopram 20mg once a day, lithium 300mg thrice a day, and olanzapine 15mg once a day. In the ED, a computed tomography (CT) of the head revealed no acute intracranial abnormalities. CT of the cervical spine was negative for any fracture. Vitals were significant for a maximum temperature of 102°F and blood pressure of 179/77. Laboratory measurements obtained were significant for a lithium level of 2.5mmol/L, white blood cell count of 15.2th/uL, and sodium of 129mmol/L. On physical examination, the patient was alert and oriented only to self (AOx1), drowsy in appearance with a mild tremor in the upper extremities, mainly on the hands. Her lithium medication was discontinued, and she was started on normal saline at 75mL/hr. The following day, her lithium level had normalized, but she was still AOx1 and tremulous. A clonus could be elicited on her feet, and she had deep tendon hyperreflexia along with muscle rigidity on both her ankles. It was at this point that we realized that she had serotonin syndrome, and cyproheptadine was initiated. For the next two nights, she became hyperthermic with a max temperature of 102.5°F. On the third day of admission, she was alert and oriented to person, place, and time (AOx3) for the first time and ceased to spike any more fevers. Cyproheptadine was continued due to the continued presence of mild tremors and an incitable clonus. However, by the fourth day, they had resolved, and the medication was discontinued. The diagnosis of serotonin syndrome at first eluded us due to the lack of agitation that is commonly seen as a presenting symptom. This was masqueraded by the concomitant presence of lithium toxicity.

## Discussion

**Figure 1:**
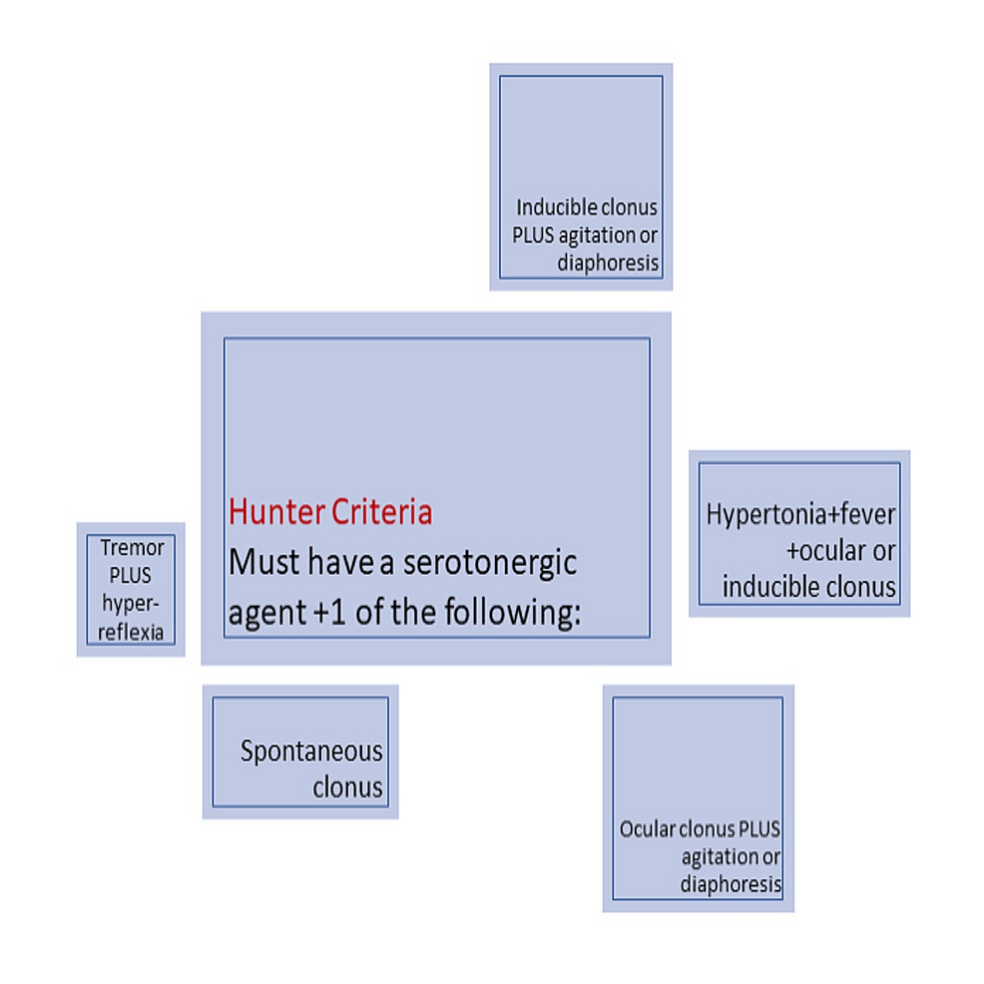
Hunter Criteria

Serotonin (5-HT) is a neurotransmitter that contains neurons in the raphe nuclei, central grey nuclei, and the medulla [1]. They play an important role in the sleep/wakefulness cycles, mood, emotional behavior, and thermoregulation [1,2]. Serotonin syndrome is the result of overstimulation of 5-HT1A receptors by serotonergic agents such as selective serotonin reuptake inhibitors (SSRIs), tricyclic antidepressants (TCAs), monoamine oxidase inhibitors (MAOI), among others [3]. It is an adverse drug reaction characterized by the triad of altered mental status, autonomic dysfunction, and neuromuscular abnormalities. Mental status changes can include agitation, confusion, and delirium. Autonomic dysfunction signs can include hyperthermia, hypertension, and tachycardia. Neuromuscular abnormalities can present with hyperreflexia, rigidity, and myoclonus [4]. A proper physical exam is very important in identifying most of these signs and symptoms, with the most common of these being deep tendon hyperreflexia and inducible or spontaneous muscle clonus. Even though diagnosis can be made on clinical findings alone, some experts recommend using the Hunter toxicity criteria decision rules; to fulfill such criteria, one must have to have taken a serotonergic agent and meet one of the criteria seen in Figure 1 [5].

Management of serotonin syndrome consists of discontinuing the offending agent, the initiation of supportive therapy to help stabilize the vital signs, administration of benzodiazepines for sedation, and the use of anti-serotonergic agents. When trying to control hyperthermia, it is important to realize that the elevated temperature is not being caused by an elevation in the temperature set point in the hypothalamus, but rather it is due to increased muscular activity. In these instances, the use of antipyretics is not indicated. In agitated patients, benzodiazepines are used for sedation, which helps control agitated patients, thus improving the blood pressure and elevated heart rate caused by the agitation. When vitals and/or agitation remain uncontrolled, the use of cyproheptadine, a histamine-1 receptor antagonist with nonspecific 5-HT1A and 5-HT2A antagonistic properties, is recommended. Prognosis is very good as long as the syndrome is identified early in its course and treated accordingly. Even though lithium toxicity can present with neuromuscular excitability in the form of irregular coarse tremors and fasciculations, it can also present with sluggishness and confusion [6]. If the latter symptoms are present from lithium toxicity along with serotonin syndrome, then identifying the problem can be more challenging.

## Conclusions

The presentation of serotonin syndrome can be masked due to the presence of other concomitant conditions, such as lithium toxicity. Physicians must understand the pathophysiology of these conditions to get to the right diagnostic and treatment path. Here we present such a case and hope that it will remind physicians of the importance of a good history and physical exam.
